# Mineralocorticoid Receptor Antagonists in Diabetic Kidney Disease

**DOI:** 10.3389/fphar.2021.754239

**Published:** 2021-10-28

**Authors:** Daiji Kawanami, Yuichi Takashi, Yoshimi Muta, Naoki Oda, Dai Nagata, Hiroyuki Takahashi, Makito Tanabe

**Affiliations:** Department of Endocrinology and Diabetes Mellitus, Fukuoka University School of Medicine, Fukuoka, Japan

**Keywords:** diabetic kidney disease, diabetic nephropathy, aldosterone, mineralocorticoid receptor (MR), mineralocorticoid receptor antagonist (MRA)

## Abstract

Diabetic kidney disease (DKD) is a major cause of end-stage kidney disease (ESKD) worldwide. Mineralocorticoid receptor (MR) plays an important role in the development of DKD. A series of preclinical studies revealed that MR is overactivated under diabetic conditions, resulting in promoting inflammatory and fibrotic process in the kidney. Clinical studies demonstrated the usefulness of MR antagonists (MRAs), such as spironolactone and eplerenone, on DKD. However, concerns regarding their selectivity for MR and hyperkalemia have remained for these steroidal MRAs. Recently, nonsteroidal MRAs, including finerenone, have been developed. These agents are highly selective and have potent anti-inflammatory and anti-fibrotic properties with a low risk of hyperkalemia. We herein review the current knowledge and future perspectives of MRAs in DKD treatment.

## Introduction

Diabetic kidney disease (DKD) is a leading cause of end-stage kidney disease (ESKD) (Tuttle et al., 2014) and clarifying the precise mechanisms responsible for DKD is becoming an urgent problem worldwide. Based on recent advances, sodium glucose transporter (SGLT) 2 inhibitors and glucagon-like peptide 1 (GLP-1) receptor agonists are attracting attention (Palmer et al., 2021; Sattar et al., 2021); however, standard care for DKD in type 1 diabetes (T1D) and type 2 diabetes (T2D) for the past two decades has been renin-angiotensin aldosterone system (RAS) blockade using angiotensin-converting enzyme inhibitors (ACEis) or angiotensin II receptor blockers (ARBs) (Frimodt-Moller et al., 2020). These drugs can improve the systemic blood pressure as well as intraglomerular pressure, thereby reducing albuminuria in DKD (Persson et al., 2016).

Aldosterone exerts its classical action of sodium absorption and potassium excretion by binding to mineralocorticoid receptor (MR) at the distal nephron (Good, 2007). Mounting evidence indicates the localization of MR in various cell types, with its expression regulated by an aldosterone-independent mechanism (Frimodt-Moller et al., 2020).

In DKD, overactivation of MR has been implicated as a driver of inflammation and fibrosis. Long-term administration of ACEis and ARBs paradoxically increases aldosterone levels. This phenomenon is known as aldosterone breakthrough and can cause renal injury (Goenka et al., 2019). These findings indicate that RAS blockade is ideal for DKD, but hyperkalemia remains a major concern associated with the combination of ACEis/ARBs and MR antagonists (MRAs) (Epstein, 2016). A previous meta-analysis demonstrated that, in CKD patients with proteinuria, the addition of MRAs to RAS inhibitors significantly reduced the blood pressure and proteinuria but increased the risk of hyperkalemia (Bolignano et al., 2014; Currie et al., 2016).

Recently, the Finerenone in Reducing Kidney Failure and Disease Progression in Diabetic Kidney Disease (FIDELIO-DKD) (Bakris et al., 2020) and the Finerenone in Reducing Cardiovascular Mortality and Morbidity in Diabetic Kidney Disease (FIGARO-DKD) (Pitt et al., 2021) showed the beneficial effects of finerenone, a selective, nonsteroidal MRA, on DKD without an increased risk for hyperkalemia. This result has suggested new possibilities regarding the significance of MRA use in DKD.

We herein review the current knowledge and future perspectives concerning the use of MRAs for DKD.

## The Renin-Angiotensin-Aldosterone System in Diabetic Kidney Disease

Circulating RAS pathway is regulated by a form of angiotensin I (Ang I), obtained from angiotensinogen, that is mediated by renin (Dostal and Baker, 1999). Angiotensin II (Ang II) binds to the AT_1_ receptor (AT_1_R) in vascular smooth muscle cells and renal tubules and then induces vasoconstriction and sodium absorption, leading to an increase in blood pressure (Mehta and Griendling, 2007). Ang II also binds to the AT_1_R in the adrenal gland, thereby promoting aldosterone production (Forrester et al., 2018). Aldosterone production is mediated by aldosterone synthase, an enzyme encoded by CYP11B2, in the adrenal cortex, and this process is stimulated by Ang II (Hattangady et al., 2012). Aldosterone binds to MR, and the bound MR translocates into the nucleus and then initiate the transcription of target genes (Gomez-Sanchez and Gomez-Sanchez, 2014).

Classical and non-classical tissue expression of MR have been demonstrated. Classical MR expression is observed in the epithelium of the collecting duct, an aldosterone-sensitive distal nephron (Shibata et al., 2013). Although aldosterone and glucocorticoid have equal affinity to MR, the specificity of aldosterone signaling is protected by 11*β*-hydroxysteroid dehydrogenase type 2 (11*β*-HSD2) (Funder, 2010). However, MR activation in non-classical tissues occur in various glomerular cell types, including mesangial cells, podocytes, and endothelial cells, independent of 11*β*-HSD2 (Brem and Gong, 2015). It is worthy to note that glucocorticoid receptor (GR) also plays an important role in the development of DKD. Deletion of endothelial GR has been shown to accelerate renal fibrosis in diabetic mice by dysregulated cytokine and chemokine reprogramming, augmented Wnt signaling and suppression of fatty acid oxidation (Srivastava et al., 2021b). Interestingly, deletion of podocyte GR has been shown to upregulate Wnt signaling and disrupt fatty acid metabolism in diabetic mice, leading to enhanced glomerulosclerosis (Srivastava et al., 2021a). Mechanistically, endothelium derived from podocyte-specific GR knockout mice showed features of endothelial-to-mesenchymal transition. These findings indicate that GR plays an important role in the pathogenesis of DKD by mediating podocyte-endothelial cell crosstalk (Srivastava et al., 2021a).

Local activation of RAS in the kidney plays an important role in the pathogenesis of DKD. The local intrarenal RAS functions as a paracrine hormonal system, independent from the systemic RAS (Culver et al., 2017). It has been shown that angiotensinogen, a substrate for renin, is expressed in the kidney and plays a key role in producing local Ang II (Kobori et al., 2003). Although angiotensinogen levels in renal tissue are much lower than those in the plasma, the renal renin activity is over 1000-fold higher than the plasma renin activity (Nishiyama and Kobori, 2018). Therefore, angiotensinogen can be easily cleaved by renin to form Ang I. Accordingly, Ang I is converted to Ang II by ACE, which is abundantly expressed in the kidney (Rosivall and Navar, 1983; Casarini et al., 1997; Komlosi et al., 2003). Ang II is thought to be generated in the interstitium and proximal tubule of the kidney (Seikaly et al., 1990; Braam et al., 1993; Nishiyama and Kobori, 2018). Consistent with these observations, intrarenal levels of Ang II are 1000-fold higher than its circulating levels in plasma, suggesting that intrarenal Ang II is mainly generated from within the kidney as a paracrine hormone (Erdos and Skidgel, 1985; Nishiyama et al., 2002). In addition to intrarenal Ang II generation, circulating Ang II is internalized in the kidney through AT_1_ receptor (Gonzalez-Villalobos et al., 2008). Although angiotensinogen is predominantly expressed in the proximal tubule (Kobori et al., 2001; Lantelme et al., 2002; Kamiyama et al., 2014), several studies have shown that the glomerular angiotensinogen levels are increased under pathophysiologic conditions, including diabetes (Ohashi et al., 2010; Eriguchi et al., 2016). Under diabetic conditions, hyperglycemia may increase angiotensinogen generation and Ang II production in the kidney (Nishiyama and Kobori, 2018). Increased ang II has been shown to induce oxidative stress, inflammation, and growth factors such as TGF-*β* and vascular endothelial growth factor (VEGF), leading to the development of DKD (Bahreini et al., 2021). AT_1_R and AT_2_R have opposite functions. AT_1_R mediates inflammatory response of Ang II. In contrast, AT_2_R counteracts the effects of AT_1_R and exerts renoprotective effects by inhibiting oxidative stress, inflammation, and apoptosis (Kaschina et al., 2017).

In addition to classical RAS pathway, ACE2/Ang (1–7)/Mas axis also plays an important role in the pathogenesis of DKD (Bader, 2013). ACE2 expresses in various tissues, including kidney, heart, liver, lung, and neurons (Padda et al., 2015; Shi et al., 2015; Culver et al., 2017; Xiao and Burns, 2017). In the kidney, ACE2 is highly expressed in tubular and glomerular epithelium, vascular smooth muscle cells, the endothelium of interlobular arteries, and glomerular mesangial cells (Lely et al., 2004). ACE2 generally counteracts functions of the conventional ACE/Ang II/AT1R axis. It degrades Ang II into the vasodilator and anti-proliferative Ang 1–7. Ang 1–7 downregulates oxidative stress, inflammation, fibrosis through its receptor, MasR (Shi et al., 2015).

Importantly, local renal aldosterone production has been reported. Nishikawa et al. confirmed that human mesangial cells express both CYP11B2 and aldosterone (Nishikawa et al., 2005). They also demonstrated that mesangial aldosterone productions are induced by Ang II, glucose, and low-density lipoprotein, all of which are inhibited by atorvastatin (Nishikawa et al., 2005; Nishikawa et al., 2010). Consistent with these observations, Siragy, et al. showed that FAD286, an aldosterone synthesis inhibitor, attenuated urinary albumin excretion and downregulated the glomerular expression of transforming growth factor (TGF)-*β*, tumor necrosis factor (TNF)-*α*, nuclear factor (NF)-κB, and interleukin (IL)-6 in adrenalectomized STZ-induced diabetic rats (Siragy and Xue, 2008). Furthermore, local aldosterone production activates MR in mesangial cells, leading to TGF-*β*/Smad2-mediated fibronectin production (Lai et al., 2003).

It has been shown that the binding of aldosterone to MR results in the activation of platelet-derived growth factor receptor and epidermal growth factor receptor and the induction of PI3K/MAPK signaling, thereby promoting the proliferation of kidney fibroblasts (Huang et al., 2012). Aldosterone induces epithelial-mesenchymal transition (EMT), a key process of interstitial fibrosis, *via* MR-mediated, mitochondrial-originated, reactive oxygen species (ROS)-dependent ERK1/2 activation in renal tubular epithelial cells (Zhang et al., 2007). Aldosterone has been shown to stimulate fibronectin production from renal fibroblasts by MR-dependent JNK/c-jun phosphorylation as well as MR-independent Src-mediated IgF1-R and subsequent ERK1/2 activation (Chen et al., 2013). These data suggest that aldosterone induces extracellular matrix production and renal fibrosis in MR-dependent and MR-independent fashions.

Taken together, these findings indicate that increased intrarenal RAS activation plays a key role *via* the MR signaling pathway in the development of DKD.

## Regulation of Mineralocorticoid Receptor and Excess Mineralocorticoid Receptor Activation in Diabetic Kidney Disease

Cloning of human MR was reported in 1987 (Arriza et al., 1987). MR is a steroid nuclear receptor family member. Aldosterone, cortisol, and progesterone bind to MR with the same affinity, although progesterone acts as an MR antagonist (Vodosek Hojs et al., 2021). Unbound MR is localized in the cytosol and is bound with its chaperones, such as Hsp90. Upon binding to ligands, the chaperones are dislocated, and dimerization of the receptor occurs (Butterworth, 2021). Ligand-bound and activated MRs translocate into the nucleus and initiate transcription by binding to hormone response elements (HREs) of target genes (Cole and Young, 2017).

Systemic MR-deletion in mice was shown to result in renal salt wasting, hyperkalemia, and increased plasma renin and aldosterone levels, leading to death at one to two weeks after birth (Berger et al., 1998). Recently, a study utilizing nephron-specific MR-deleted mice was reported. In these mice, the epithelial Na^+^ channel (ENaC) activity was abolished in the cortical collecting duct but not in the distal convoluted tubule, indicating that MR determines ENaC activity in the cortical collecting duct but to a lesser degree in the distal convoluted tubule (Wu et al., 2020). Regulation of ENaC by MR activation occurs *via* activation of serum and glucocorticoid regulated kinase-1 (SGK1) (Belden et al., 2017), an effector kinase. The TGF-*β*/SGK1 pathway has been shown to play a crucial role in the fibrotic response in the kidney (Cheng et al., 2010; Miao et al., 2019). MR activation has been shown to occur *via* an aldosterone-independent mechanism. Several factors, such as PKA, the small guanosine triphosphatase (GTPase) RAS-related C3 botulinus toxin substrate 1 (Rac1), and ubiquitin conjugating enzymes, are involved in MR activation (Parker et al., 2018).

Crosstalk between Rac1 and MR has been extensively investigated, as Rac1 activates MR in a ligand-independent fashion (Nagase and Fujita, 2011). Shibata et al. demonstrated that Rac1 activation in podocytes resulted in proteinuria without affecting systemic aldosterone status (Shibata et al., 2008). They also demonstrated that Rac1 activation is essential for salt-sensitive hypertension *via* a MR-dependent pathway (Shibata et al., 2011). Oxidative stress has been shown to induce MR activation in a ligand-independent, Rac1-depenent manner (Kawakami-Mori et al., 2012; Nagase et al., 2012). It has been shown that Rac1 is activated by high glucose levels in cultured mesangial cells, and MR activation is attenuated by pharmacologic and dominant-negative-mediated Rac1 inhibition, indicating that Rac1 plays an important role in MR activation under diabetic conditions (Yoshida et al., 2014). Consistent with this observation, it was confirmed that EHT 1864, a Rac1 inhibitor, attenuated albuminuria along with reducing pathological glomerular changes in KK-A^y^ mice (Yoshida et al., 2014). Rac1 activation is associated with dysregulation of podocyte actin cytoskeleton dynamics, leading to the development of glomerulosclerosis (Blattner et al., 2013). Hirohama et al. reported that the Rac1 inhibitor NSC23766 attenuated high-salt-induced albuminuria in T2D db/db mice with uninephrectomy (Hirohama et al., 2021). They observed Rac1 activation in podocytes and SGK1 activation in the renal cortex in these mice, all of which were downregulated by Rac1 inhibition, suggesting the involvement of the Rac1-MR signaling pathway in DKD (Hirohama et al., 2021).

**FIGURE 1:**
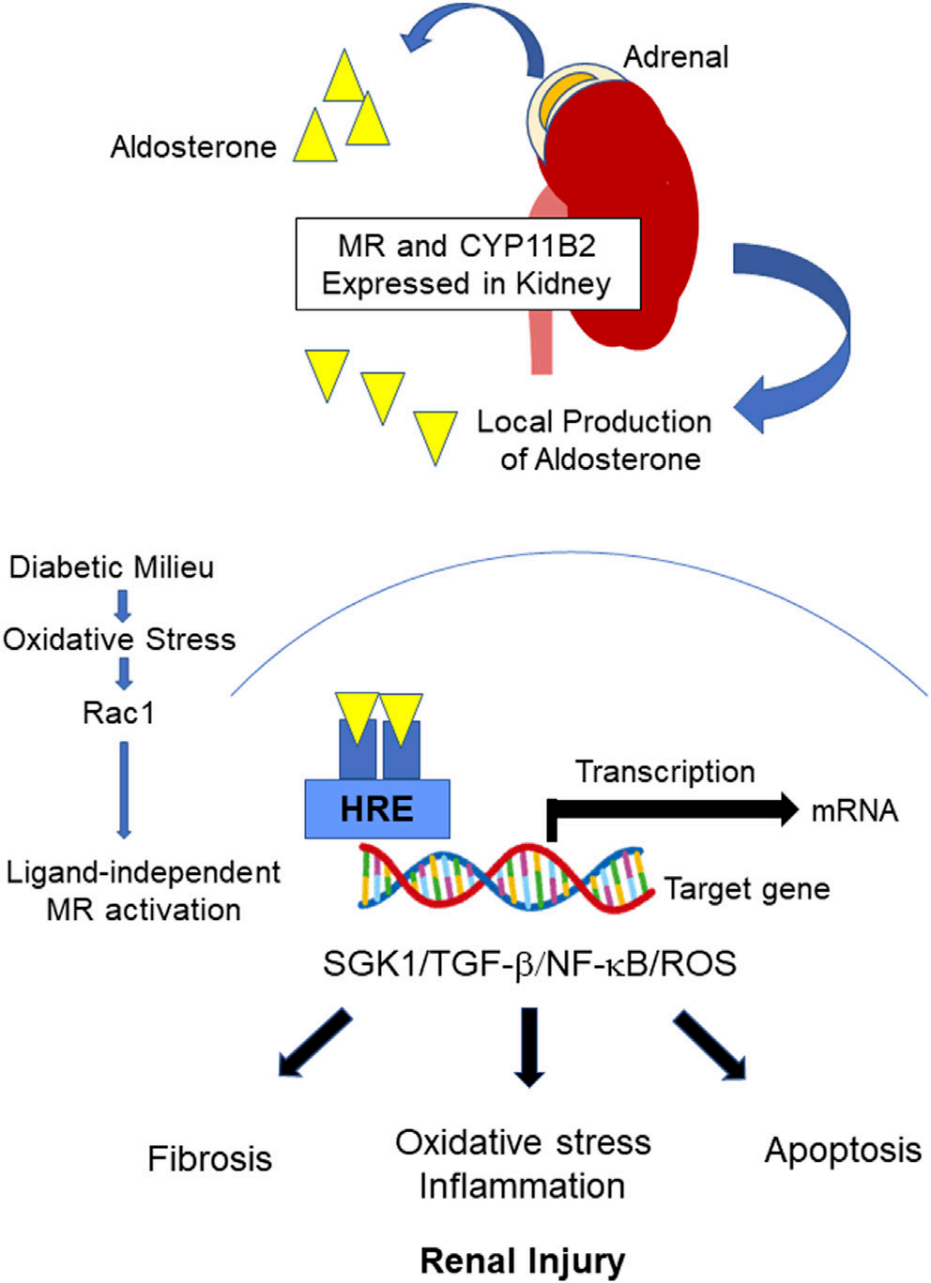
Regulation of MR in DKD. Upon the binding of aldosterone to MR, the bound MR translocates into the nucleus and binds to the HRE of the target gene to initiate transcription. CYP11B2 is expressed in the kidney, and local aldosterone production occurs in DKD, leading to MR overactivation. MR overactivation stimulates SGK1 and induces subsequent inflammation/oxidative stress, apoptosis, and fibrotic process. Furthermore, ligand-independent MR activation by Rac1 has been reported. MR: mineralocorticoid receptor, DKD: diabetic kidney disease, HRE: Hormone responsive element, SGK1: Serum and glucocorticoid-regulated kinase 1, TGF-*β*: transforming growth factor beta, NF-κB: Nuclear factor κB, ROS: reactive oxygen species, Rac1: The small guanosine triphosphatase (GTPase) RAS-related C3 botulinus toxin substrate 1.

The regulation of MR in DKD is shown in Figure 1. Clinically, the standard of care in DKD have been blockade of RAS with ACEis or ARBs on top of glucose management (Frimodt-Moller et al., 2020). As mentioned previously, aldosterone breakthrough is seen during treatment with RAS inhibitors. Furthermore, MR is overactivated as described above. Therefore, MR has been implicated as an important therapeutic target in DKD patients who are taking ACEi and/or ARBs (Frimodt-Moller et al., 2020). Steroidal MRAs have been clinically used. Recently, nonsteroidal MRAs have been developed (Al Dhaybi and Bakris, 2020; Wada et al., 2021; Wan et al., 2021). Differences in steroidal and nonsteroidal MRAs are shown in Table 1. Significance of MRAs use in DKD treatment will be described in following sections.

**TABLE 1:** Differences between steroidal and nonsteroidal MRAs. Each MRA has different half-life and tissue distribution (Lentini et al., 2016; Agarwal et al., 2021; Wan et al., 2021). Both of those drugs can cause hyperkalemia. However, nonsteroidal MRAs are thought to have less risk of hyperkalemia because of their equal tissue distribution in kidney and heart (Agarwal et al., 2021).

	Spironolactone	Eplerenone	Esaxerenone	Finerenone
Class	Steroidal	Steroidal	Nonsteroidal	Nonsteroidal
Half-life	14–16 h	4–6 h	18–25 h	2 h
Tissue distribution	Kidney > Heart	Kidney > Heart	Kidney = Heart	Kidney = Heart
Side effects	Hyperkalemia	Hyperkalemia	Hyperkalemia	Hyperkalemia
	Hormonal (gynecomastia impotence menstrual irregularities)		(Lower risk compared to steroidal MRAs)	

## Steroidal Mineralocorticoid Receptor Antagonist

### Spironolactone

Spironolactone is a first-developed MRA, and its structure is based on progesterone, which has an antagonistic effect on MR (Yang and Young, 2016). Spironolactone binds to MR at the same site of aldosterone (Vodosek Hojs et al., 2021). As spironolactone is not a selective MRA and has an agonistic effect on progesterone receptor as well as an antagonistic effect on androgen receptor, its use causes gynecomastia (Funder, 2019). The effects of spironolactone on DKD have been widely investigated.

• Basic Studies

Spironolactone has been shown to attenuate aldosterone-induced apoptosis in cultured mesangial cells by activating the Wnt signaling pathway (Zhu et al., 2013). Spironolactone has been shown to attenuate high-glucose-induced podocyte apoptosis (Lee et al., 2009). Consistent with this observation, spironolactone has been shown to prevent high-glucose-induced podocyte injury by reducing SGK1 and NADPH oxidase activity (Toyonaga et al., 2011).

Fujisawa et al. demonstrated that spironolactone attenuated the MR activation-mediated plasminogen activator inhibitor (PAI)-1 expression, TGF-*β* expression, macrophage infiltration, and renal fibrosis in streptozotocin (STZ)-induced diabetic rats (Fujisawa et al., 2004). Interestingly, Taira et al. showed that spironolactone prevented the induction of renal CYP11B2 mRNA expression in uninephrectomized STZ-induced diabetic rats (Taira et al., 2008), suggesting that spironolactone inhibits local aldosterone production.

Integrin *β*1 and *β*3 play important roles in maintaining podocyte structure. Li et al. demonstrated that spironolactone prevented podocyte structural destruction by decreasing integrin *β*1 and increasing integrin *β*3 under high-glucose conditions (Li et al., 2015). Furthermore, spironolactone has been shown to ameliorate podocytic adhesive capacity by restoring the integrin *α*3 expression (Lin et al., 2010). Spironolactone has also been shown to promote autophagy by inhibiting the PI3K/AKT/mTOR signaling pathway and reducing the mechanical stress-induced podocyte damage (Li et al., 2016). Consistent with this observation, spironolactone has been shown to attenuate glomerulosclerosis and interstitial fibrosis by promoting autophagy in diabetic rats induced by a high-fat diet and low-dose STZ. A mechanistical analysis revealed that spironolactone prevented podocyte loss along with increasing Beclin1 and anti-LC3B, markers of autophagy (Dong et al., 2019).

Spironolactone has been shown to attenuate aldosterone-mediated renal tight junction (TJ) injury in T1D rats (Molina-Jijon et al., 2017). In that study, the administration of spironolactone to STZ-induced diabetic rats prevented the downregulation of TJ proteins, such as claudin-2, claudin-4, claudin-5, and claudin-8, as well as occludin in the glomeruli and proximal and distal tubules. These observations were mediated by reduced oxidative stress through the spironolactone-induced inhibition of SGK1 (Molina-Jijon et al., 2017).

Several studies have demonstrated the beneficial synergistic effects of spironolactone plus ARBs on DKD in diabetic animal models (Hofni et al., 2014; Lee et al., 2011).

• Clinical Studies

Several RCTs to examine the add-on effects of spironolactone on DKD have been reported. Spironolactone monotherapy (25 mg daily) has been shown to reduce albuminuria in T2D patients with DKD as effectively as combination therapy of spironolactone 25 mg plus losartan 25 mg for 3 months (Makhlough et al., 2014). At the end of that study, both groups showed comparable rates of treatment success (70% in the spironolactone group and 83.3% in the spironolactone plus losartan group), defined as a more than 50% reduction in albuminuria (Makhlough et al., 2014). Mehdi et al. described albuminuria in T1D and T2D patients who all received lisinopril (80 mg daily). In that study, participants were randomly assigned to receive placebo, losartan (100 mg daily), or spironolactone (25 mg daily) for 48 weeks. The UACR (urinary albumin-creatinine ration) was decreased by 34.0% (95% confidence interval [CI]: −51.0% to −11.2%, *p* = 0.007) in the spironolactone group and by 16.8% (95% CI: −37.3% to +10.5%, *p* = 0.20) in the losartan group (Mehdi et al., 2009). Esteghamati et al. reported that 18-months administration of spironolactone (25 mg daily) plus losartan (50–100 mg daily) was more effective in reducing albuminuria in DKD patients than the dual combination of ACEis and ARBs (Esteghamati et al., 2013). Kato et al. demonstrated that 8-weeks administration of spironolactone (25 mg daily) with conventional RAS inhibitors significantly reduced albuminuria by 33% (95% CI: 22–54; *p* = 0.0002) in Japanese T2D patients, independent of blood pressure reductions (Kato et al., 2015). A meta-analysis that investigated the effects of spironolactone added to preexisting anti-hypertensive treatment on DKD progression has been reported. That study included 16 randomized control trials (RCTs) in which spironolactone was added to existing therapies, such as ACEis and/or ARBs. The combination of spironolactone with RAS inhibitors resulted in a reduction in blood pressure and proteinuria in patients with DKD (Hou et al., 2015).

Recently, the proteomic prediction and renin angiotensin aldosterone system inhibition prevention of early diabetic nephropathy in type 2 diabetic patients with normoalbuminuria (PRIORITY) study investigated whether or not spironolactone could prevent the incidence of microalbuminuria in T2D patients (defined as a urinary proteomic pattern) (Tofte et al., 2020). After a median follow-up of 2.51 years, spironolactone failed to prevent progression to microalbuminuria in T2D patients (Tofte et al., 2020). It is worth noting that spironolactone use has been shown to be associated with worsening of glucose metabolism and increased HbA1c and cortisol levels (Yamaji et al., 2010; Zhao et al., 2016).

### Eplerenone

Eplerenone is a steroidal MRA that has a higher selectivity for MRAs than spironolactone. The beneficial effects of eplerenone have been extensively investigated in patients with hypertension (Weinberger et al., 2002) and heart failure (Zannad et al., 2011). In addition, eplerenone has been shown to reduce morbidity and mortality in patients with left ventricular dysfunction after myocardial infarction (Pitt et al., 2003) *via* its favorable effects on fibrosis and cardiovascular remodeling (Agarwal et al., 2021).

• Basic Studies

The administration of eplerenone has been shown to attenuate renal fibrosis by inhibiting the TGF-*β* and collagen IV expression in hypertensive diabetic rats (Lian et al., 2012). Nagase et al. demonstrated that eplerenone reduced proteinuria by preventing podocyte injury and glomerulosclerosis in Dahl salt-hypertensive rats (Nagase et al., 2006). The inhibition of oxidative stress by eplerenone appears to be involved in this observation, as Shibata et al. reported that eplerenone attenuates proteinuria in uninephrectomized rats with aldosterone infusion by downregulating SGK1 and subsequently increasing the NADPH oxidase activity in podocytes (Shibata et al., 2006). Nishiyama et al. showed that eplerenone enhanced the antiproteinuric effect of an ARB by inhibiting podocyte injury in OLETF rats, independent of blood-pressure-lowering effects (Nishiyama et al., 2010). They also demonstrated that eplerenone inhibits mesangial cell proliferation, which is mediated by high-glucose-induced big mitogen-activated protein kinase 1 (BMK1) activation (Liu et al., 2010).

These findings indicate that eplerenone exerts renoprotective effects, independent of reductions in blood pressure.

• Clinical Studies

Epstein et al. reported that eplerenone reduced albuminuria in T2D patients (Epstein et al., 2006). In this RCT, 286 participants who were taking enalapril (20 mg daily) were allocated to the placebo group, eplerenone 50 mg group, and eplerenone 100 mg group. After 12 weeks, the UACR was reduced by 7.4, 41.0, and 48.4% in the placebo, eplerenone 50 mg, and eplerenone 100 mg groups, respectively (Epstein et al., 2006). Importantly, there were no significant changes in the incidences of sustained or severe hyperkalemia among the three groups (Epstein et al., 2006). Tsuboi et al. demonstrated that eplerenone reduced proteinuria in non-diabetic CKD patients who were treated with RAS inhibitors. They found that the administration of eplerenone (25–50 mg daily) for 12 months resulted in a 38% reduction in urinary protein excretion (Tsuboi et al., 2012). Recently, Mokadem et al. performed a RCT to investigate the effect of eplerenone on albuminuria in T2D patients with hypertension. In that study, 75 patients were randomly allocated to the ramipril group (10 mg daily) group, eplerenone (50 mg daily) group, and eplerenone (50 mg)/ramipril (10 mg) combination group. After a 24-weeks follow-up period, ramipril and eplerenone monotherapy had significantly reduced the UACR compared with the baseline, and the eplerenone/ramipril combination group showed an even greater reduction in the UACR than the ramipril and eplerenone monotherapy groups (El Mokadem et al., 2020).

## Non-steroidal Mineralocorticoid Receptor Antagonist

### Finerenone

Steroid hormone receptors, including MR, have been shown to interact with cofactors that affect gene transcription, and steroidal MRAs can interact with cofactors, leading to their functioning as partial MR agonists (Agarwal et al., 2021). Therefore, the actions of nonsteroidal MRAs differ from those of steroidal MRAs, such as spironolactone and eplerenone, as nonsteroidal MRA blocks the MR as a bulky and passive antagonist (Amazit et al., 2015). Finerenone has been developed as a potent and selective nonsteroidal MRA. Finerenone inhibits cofactor recruitment to the MR in the absence of aldosterone and functions as an inverse agonist. In addition, the gene regulation profile by finerenone differs from that for steroidal MRAs (Grune et al., 2016). Finerenone has more potent antifibrotic activity than eplerenone. For instance, differential MR cofactor modulation is proposed to be associated with finerenone-specific amelioration of tenascin X (TNX), an MR target gene that is a crucial regulator of fibrosis (Grune et al., 2018).

In addition, finerenone is expected to carry a lower risk of hyperkalemia than steroidal MRAs. One possible mechanism involves its tissue distribution. A study using [^14^C]-labelled finerenone demonstrated a balanced kidney-heart distribution, although spironolactone and eplerenone showed a dominant distribution in the kidney compared with the heart (Platt and Pauli, 1972; Kolkhof et al., 2014; Agarwal et al., 2021). These differences may affect the sodium and potassium balance (Kolkhof et al., 2014). The pharmacokinetics of finerenone are also different from those of spironolactone and eplerenone, as finerenone has no active metabolites and a short half-life (2 h) (Lentini et al., 2016). In contrast, spironolactone is a prodrug that has multiple active metabolites with long half-lives (14–16 h), and eplerenone has no active metabolites with a half-life of 4–6 h (Gardiner et al., 1989; Cook et al., 2003; Veneti and Tziomalos, 2021).

• Basic Studies

Kolkhof et al. demonstrated that finerenone prevented organ damage to the heart and kidney in deoxycorticosterone acetate-/salt-induced hypertensive rats and rats with chronic heart failure after coronary artery ligation (Kolkhof et al., 2014). Importantly, they found that finerenone exerted cardiorenoprotective effects independent of its blood pressure reduction (Kolkhof et al., 2014). Combination therapy of empagliflozin and finerenone has also been shown to prevent proteinuria as well as cardiac and kidney fibrosis in hypertensive rats (Kolkhof et al., 2021).

Finerenone has been shown to prevent tubular injury in a rat ischemic acute kidney injury model [ischemia/reperfusion (IR)]. In a previous study, the effects of finerenone on the progression of acute kidney injury (AKI) to CKD was evaluated at 4 months after IR (Lattenist et al., 2017). Finerenone significantly attenuated tubulointerstitial fibrosis and the TGF-*β* expression by downregulating the oxidative stress in rats receiving IR (Lattenist et al., 2017). Finerenone has also been shown to reduce oxidative stress by inhibiting Rac1 activation and the subsequent MR signaling pathway in vascular smooth muscle cells (Barrera-Chimal et al., 2017). Myeloid MR plays an important role in IR-mediated renal fibrosis.

M2-antiinflamatory markers are reportedly increased in macrophages from finerenone-treated and myeloid MR-deficient mice. Furthermore, the inflammatory population of CD11b^+^, F4/80^+^, Ly6C^high^ macrophages was also reduced upon myeloid MR inhibition by finerenone. From a mechanistic standpoint, finerenone promoted IL-4 receptor-dependent signaling, thereby facilitating M2 polarization in macrophages (Barrera-Chimal et al., 2018).

Le Billan et al. investigated the effects of spironolactone and finerenone on the aldosterone-induced transcriptome of a human renal cell line stably expressing MR. They found similar gene expression profiles in both MRAs, but finerenone exerted more efficient antagonism on some aldosterone-induced genes (Le Billan et al., 2021).

• Clinical Studies

Phase 2 trials of finerenone have been reported, including the mineralocorticoid receptor antagonist tolerability study (ARTS), ARTS-Heart Failure (ARTS-HF), and ARTS-Diabetic Nephropathy (ARTS-DN).

ARTS is an RCT that assessed changes in the serum potassium levels by finerenone as a primary endpoint in patients with HFrEF and mild or moderate CKD (Pitt et al., 2013). This study consisted of two parts. In part A, the safety and tolerability of finerenone at 2.5, 5, or 10 mg once daily was assessed in 65 patients with HFrEF and mild CKD [estimated glomerular filtration rate (eGFR) 60–90 ml/min/1.73 m^2^]. In part B, the effects of finerenone at 2.5, 5, or 10 mg once daily or 5 mg twice daily were compared with those of placebo and open-label spironolactone (25 or 50 mg/day) in 392 patients with HFrEF and moderate CKD (eGFR 30–60 ml/min/1.73 m^2^) (Pitt et al., 2013). After a follow-up period of 28 days, finerenone at all doses was associated with significantly smaller increases in serum potassium levels and lower incidences of hyperkaliemia and eGFR decline than spironolactone 50 mg daily. Finerenone also decreased the levels of B-type natriuretic peptide (BNP), amino-terminal proBNP, and albuminuria at least as much as spironolactone (Pitt et al., 2013). Taken together, these findings suggest that finerenone (5 or 10 mg/day) was as efficient at lowering albuminuria and cardiac biomarkers as spironolactone (25 or 50 mg/day) with a smaller risk of hyperkalemia.

The primary endpoint of ARTS-HF was the proportion of patients with a >30% decline in NT-proBNP from baseline (Filippatos et al., 2016). In that study, 1,066 patients with HFrEF who have T2D and/or CKD (eGFR > 30 ml/min/1.73 m^2^ among patients with T2D and 30–60 ml/min/1.73 m^2^ among those without T2D) were randomly assigned to once-daily finerenone (2.5, 5, 7.5, 10, or 15 mg, uptitrated to 5, 10, 15, 20, or 20 mg, respectively, on day 30) or eplerenone (25 mg every other day, increased to 25 mg once daily on day 30, and to 50 mg once daily on day 60) and were followed for 90 days (Filippatos et al., 2016). Finerenone was well tolerated and induced a ≥30% decrease in NT-proBNP levels in a similar proportion of patients to eplerenone (Filippatos et al., 2016).

In ARTS-DN, a total of 823 T2D patients with albuminuria (UACR ≥ 30 mg/g), and eGFR >30 ml/min/1.73 m^2^ who were treated with RAS inhibitors were enrolled (Bakris et al., 2015). Patients were randomly allocated to receive either finerenone (1.25, 2.5, 5, 7.5, 10, 15, and 25 mg daily) or a placebo and were followed for 90 days. The primary endpoint of this study was the change in the UACR from baseline. Finerenone reduced the UACR in a dose-dependent fashion compared to placebo as follows: 0.79 (90% CI: 0.68–0.91; *p* = 0.004) in the 7.5 mg group, 0.76 (90% CI: 0.65–0.88; *p* = 0.001) in the 10 mg group, 0.67 (90% CI: 0.58–0.77; *p* < 0.001) in the 15 mg group, and 0.62 (90% CI, 0.54–0.72; *p* < 0.001) in the 20 mg group (Bakris et al., 2015). The reduction in albuminuria was not associated with changes in the blood pressure or eGFR, suggesting that renoprotection by finerenone was independent of the hemodynamic effects (Bakris et al., 2015).

FIDELIO-DKD was performed to assess whether or not finerenone slows CKD progression and reduces cardiovascular morbidity and mortality in patients with advanced CKD and T2D (Bakris et al., 2020). A total of 5,734 individuals with a UACR of 30–300 mg/g, eGFR 25–60 ml/min/1.73 m^2^, and a history of diabetic retinopathy or a UACR of 300–5,000 mg/g and an eGFR of 25–75 ml/min/1.73 m^2^ were enrolled. All participants were taking RAS inhibitors. Participants were randomly assigned in a 1:1 ratio to receive finerenone or placebo. Doses of finerenone were determined based on the renal function of patients. Participants with an eGFR 25–60 ml/min/1.73 m^2^ at the screening visit received an initial dose of 10 mg once daily, and those with an eGFR of ≥60 ml/min/1.73 m^2^ at the screening visit received an initial dose of 20 mg once daily. The primary outcome was a composite of kidney failure, a sustained decrease of at least 40% in the eGFR from baseline over a period of at least 4 weeks, or death from renal causes. Kidney failure was defined as ESKD or an eGFR <15 ml/min/1.73 m^2^. The key secondary composite outcome was death from cardiovascular causes, nonfatal myocardial infarction, nonfatal stroke, or hospitalization for heart failure (Bakris et al., 2020). After a median follow-up period of 2.6 years, a primary composite outcome occurred in 17.8% of the finerenone group and 21.1% of the placebo group [hazard ratio (HR) 0.82, 95% CI: 0.73–0.93, *p* = 0.001]. A key secondary outcome event occurred in 13.0% in the finerenone group and 14.8% in placebo group (HR 0.86; 95% CI: 0.75–0.99, *p* = 0.03). The frequency of adverse events was similar in both groups (Bakris et al., 2020). The incidence of hyperkalemia-related discontinuation of the trial regimen was higher with finerenone than with placebo (2.3 and 0.9%, respectively).

Previous studies have demonstrated that aldosterone upregulation and MR overactivation are involved in structural cardiac remodeling and the pathophysiology of atrial fibrillation (Reil et al., 2012; Lavall et al., 2014). A sub-analysis of FIDERIO-DKD investigated the effect of finerenone on new-onset atrial fibrillation or flutter (AFF) and cardiorenal effects by the history of AFF. In FIDERIO-DKD, 461 (8.1%) of 5,674 participants had a history of AFF. The incidence rate of AFF was significantly lower in the finerenone group than in the placebo group (HR: 0.71, 95% CI: 0.53–0.94, *p* = 0.016) (Filippatos et al., 2021). Finerenone reduced the primary and key secondary kidney and cardiovascular outcomes, irrespective of the history of AFF (Filippatos et al., 2021).

Finerenone in Reducing CV Mortality and Morbidity in Diabetic Kidney Disease (FIGARO-DKD) was a trial that assessed the efficacy of finerenone on cardiovascular and renal outcomes and its safety in T2D patients with CKD (Ruilope et al., 2019). The primary endpoint was the composite of cardiovascular death, nonfatal myocardial infarction, nonfatal stroke or hospitalization for heart failure. The key prespecified secondary endpoint was the same as the primary composite endpoint of FIDELIO-DKD (Ruilope et al., 2019). Patients included in FIGARO-DKD were T2D patients on RAS inhibitors with CKD, defined as those with a UACR of 30–300 mg/g and an eGFR 25–90 ml/min/1.73 m^2^ (CKD stage 2–4) or a UACR of ≥300 mg/g and an eGFR ≥60 ml/min/1.73 m^2^ (CKD stage 1–2) (Pitt et al., 2021), indicating that FIGARO-DKD included more patients with earlier-stage CKD and T2D than FIDELIO-DKD. In FIGARO-DKD, a total of 7,437 patients were allocated to placebo or finerenone group and followed-up for 3.4 years, a primary outcome event was significantly reduced in finerenone group (HR 0.87, 95%CI: 0.76 to 0.98, *p* = 0.03), with the benefit driven primarily by a lower incidence of hospitalization for heart failure (HR 0.71, 95% CI: 0.56–0.90). The secondary composite outcome occurred in 9.5% in the finerenone group and in 10.8% in the placebo group (HR 0.87, 95% CI, 0.76–1.01) (Pitt et al., 2021). These findings demonstrate that finerenone has cardiorenal protective effects in T2D patients with DKD.

### Apararenone

Apararenone is a highly selective and long-acting MRA. To our knowledge, no preclinical studies have investigated the cardiorenal benefits of aprarenone. A previous RCT investigating the effects of apararenone on albuminuria in T2D patients with UACR ≥50 mg/g Cr showed the renal benefit (Wada et al., 2021). In that study, 293 patients were randomly assigned to the placebo group or 2.5, 5, or 10 mg apararenone groups. More than 60% of participants were taking ACEis or ARBs. After 24 weeks, apararenone significantly decreased the UACR by 62.9% (95% CI: 54.6–72.5) (2.5 mg), 50.8% (95% CI: 44.1–58.4) (5 mg), and 46.5% (95% CI: 40.4–53.5) (10 mg) compared to the placebo. The UACR remission rates at week 24 were 0.0% (placebo), 7.8% (apararenone 2.5 mg), 29.0% (apararenone 5 mg), and 28.1% (apararenone 10 mg) (Wada et al., 2021).

Another RCT investigated whether or not apararenone had beneficial effects on nonalcoholic steatohepatitis (NASH). In that study, 48 patients with NASH were randomly assigned to receive placebo or apararenone 10 mg daily and followed for 72 weeks (Okanoue et al., 2021). The percent changes in serum alanine aminotransferase (ALT) from baseline to 24 weeks, which is the primary endpoint, were −3.0% (placebo) and −13.7% (apararenone), showing no significance (*p* = 0.308) (Okanoue et al., 2021). Apararenone showed a greater reduction in fibrosis markers and better liver fibrosis scores and fibrosis-4 indices at all time points than placebo (Okanoue et al., 2021).

### Esaxerenone

Esaxerenone was developed as a potent and selective non-steroidal MRA. The Esaxerenone and Eplerenone in Patients With Essential Hypertension (ESAX-HTN) Study was performed as a phase 3 clinical trial. In this study, 1,001 Japanese hypertensive patients were randomly assigned to either esaxerenone at 2.5 or 5 mg daily or eplerenone at 50 mg and were followed for 12 weeks (Ito et al., 2020a). Esaxerenone was effective and well-tolerated with a BP-lowering activity equivalent to or better than that of eplerenone (Ito et al., 2020a). Esaxerenone has been shown to bind to MR through the MR ligand binding domain and large side chains, thereby demonstrating a high affinity and selectivity for MR (Takahashi et al., 2020). In addition, it has long a half-life of 18.6–25.1 h (Wan et al., 2021).

• Basic Study

Esaxerenone has been shown to attenuate the development of albuminuria, glomerular injury, and tubulointerstitial fibrosis more potently than losartan in Dahl salt-sensitive hypertensive rats by reducing renal oxidative stress (Li et al., 2019). Renoprotective effects of esaxerenone have been also demonstrated in KK-A^y^ mice. In a previous study, the administration of esaxerenone ameliorated albuminuria and glomerular injury, tubulointerstitial fibrosis, renal inflammation, and oxidative stress in KK-A^y^ mice with or without a high-salt diet (Bhuiyan et al., 2019). Furthermore, the combination of esaxerenone with olmesartan synergistically attenuated albuminuria in KK-A^y^ mice accompanied by reductions in the urinary excretion of podocalyxin and monocyte chemoattractant protein 1 (Arai et al., 2020). Importantly, this combination therapy did not affect the blood pressure, indicating that esaxerenone exerted renoprotective effects independent of its blood-pressure-lowering effects (Arai et al., 2020).

• Clinical Study

Esaxerenone has been shown to improve both microalbuminuria and macroalbuminuria in patients with T2D. In a short-term study, 365 hypertensive or normotensive T2D patients with microalbuminuria who are receiving RAS inhibitors were randomized to receive esaxerenone or placebo for 12 weeks. At the end of the study, esaxerenone (1.25, 2.5, and 5 mg daily) significantly reduced albuminuria in a dose-dependent manner (Ito et al., 2019).

The Esaxerenone in Patients with Type 2 Diabetes and Microalbuminuria (ESAX-DN) Study investigated the effects of esaxerenone on microalbuminuria in T2D patients who were receiving RAS inhibitors (Ito et al., 2020b). The primary endpoint was UACR remission (<30 mg/g creatinine and a ≥30% reduction from baseline on 2 consecutive occasions). In that study, 455 T2D patients with microalbuminuria were randomly assigned to the placebo or esaxerenone group (initiated at 1.25 mg and titrated to 2.5 mg daily). After a follow-up period of 52 weeks, esaxerenone showed a significantly higher rate of UACR remission than the placebo groups (22 vs 4%). The changes in UACR from baseline were -58% in the esaxerenone group and 8% in the placebo group (Ito et al., 2020b). Hyperkalemia was observed in 9% of subjects in the esaxerenone group and 2% of subjects in the placebo group (Ito et al., 2020b).

A multicenter, single-arm, open label phase III study was performed to evaluate the renoprotective effects of esaxerenone (Ito et al., 2021). A total of 56 T2D patients with macroalbuminuria (≥UACR 300 mg/g) were administrated esaxerenone (initiated at 1.25 mg and titrated to 2.5 mg daily) for 28 weeks. At the end of the study, the UACR had decreased by 54.6% (95% CI: 46.9–61.3%), and 51.8% of participants achieved a transition to microalbuminuria (<UACR 300 mg/g). Hyperkalemia occurred in 5.4% of participants. In addition, 8.9% of participants discontinued esaxerenone because of a ≥30% decrease in the eGFR (Ito et al., 2021).

Key clinical studies of nonsteroidal MRAs are summarized in Table 2.

**TABLE 2:** Key clinical trials of nonsteroidal MRAs. The renoprotective effects finerenone and esaxerenone have been reported thus far. The primary endpoint of FIDELIO-DKD was kidney composite outcome (Bakris et al., 2020). In FIGARO-DKD, the primary endpoint was cardiovascular composite outcome. FIGARO-DKD included relatively earlier stage of DKD patients compared to FIDELIO-DKD (Pitt et al., 2021).

Study name	Patients	Drugs	Results
FIDELIO-DKD (*n* = 5,734)	T2D patients with advanced CKS (UACR 30–300 mg/g eGFR 25–60 ml/min/1.73 m^2^ and a history of diabetic retinopathy or UACR 300–5,000 mg/g and an eGFR of 25–75 ml/min/1.73 m^2^)	Finerenone (10 or 20 mg) vs Placebo 2.6 years	The primary kidney composite outcome (kidney failure a sustained decrease of at least 40% in the eGFR from baseline death) was significantly reduced by finerenone (HR 0.82; 95%CI: 073–0.93)
FIGARO-DKD (*N* = 7,437)	T2D patients with UACR 30–300 mg/g and eGFR 25–90 ml/min/1.73 m^2^ (stage 2–4 CKD) or UACR 300–5,000 mg/g and an eGFR of at least 60 ml/min/1.73 m^2^ (stage 1 or 2 CKD)	Finerenone (10 or 20 mg) vs Placebo 3.4 years	The primary Cardiovascular composite outcome (Cardiovascular death nonfatal myocardial infarction, nonfatal stroke or hospitalization for heart failure) was significantly reduced by finerenone (HR 0.87; 95%CI: 076–0.98)
ESAX-DX (*n* = 455)	T2D patients with microalbuminuria	Esaxeerenone (initiated at 1.25 mg and titrated to 2.5 mg daily) vs placebo 52 weeks	The changes in UACR from baseline were-585 in esaxerenone group and 8% in placebo group

## Future Directions and Perspectives

Even with widespread use of SGLT2 inhibitors and GLP-1 receptor agonists, a substantial residual risk of DKD progression remains. Nonsteroidal MRAs potentially complement this risk. However, some clinical questions need to be addressed. First, it needs to be clarified at which stage of DKD MRA should be started. Second, it remains unclear whether monotherapy of nonsteroidal MRAs is effective on DKD. Third, it will be necessary to elucidate what kind of anti-diabetic drugs and nonsteroidal MRAs are effective in combination because a subgroup analysis of FIDELIO-DKD demonstrated that a reduction in UACR with finerenone was observed with or without baseline GLP-1 receptor agonists use (Rossing et al., 2021). To clarify these points will determine the position of nonsteroidal MRAs in DKD treatment. Currently, the development of novel therapeutic agents for DKD that target inflammation and fibrosis is in progress (Yamazaki et al., 2021). Among them, JAK/STAT inhibitors have been shown to exert renoprotective effects in patients with DKD (Brosius et al., 2016). It is important that finerenone have both renal and cardiovascular protective effects clinically that have not yet been clarified with these developing drugs. Combined effect of these drugs and nonsteroidal MRA can also be expected. Unlike SGLT2 inhibitors and incretin-based drugs, the dose of nonsteroidal MRAs can be adjusted for renoprotection without considering glucose-lowering effects in patients with DKD, which may be advantage of nonsteroidal MRAs. Furthermore, expectations for nonsteroidal MRAs go beyond cardiorenal protection. It has been shown that endothelial cell MR mediates hypertensive remodeling in cerebral arteries, leading to reduced cerebral perfusion, which can cause stroke and dementia (Diaz-Otero et al., 2017). In addition, cortical thickness of brain has been shown to be correlated negatively with the expression of MR in human (Parker et al., 2020). Finally, MR has been shown to be involved in the development of sarcopenia (Burton et al., 2011; Lee et al., 2021). These findings demonstrate potential usefulness of nonsteroidal MRAs for geriatric syndromes in patients with diabetes by their potent anti-inflammatory properties. Future studies to elucidate comprehensive effects of nonsteroidal MRAs on diabetic complications and related disorders will be interesting.

## Conclusion

As described, the advent of nonsteroidal MRAs has revolutionized the treatment for DKD. The usefulness of steroidal MRAs on DKD has been proven by experimental studies; however, the adverse effects of hyperkalemia tend to prevent its use. Potent anti-inflammatory and anti-fibrotic responses with a reduced risk for hyperkalemia can be expected by using the nonsteroidal MRA finerenone, given its high selectivity for MRA and actions as an inverse agonist. DKD is closely associated with CVD in patients with diabetes. MR overactivation is an important factor that connects these cardiorenal complications. Appropriate interventions for MR will reduce the residual risk and bring us one step closer to overcoming DKD.
